# Under Construction: One State's Approach to Creating Health Homes for Individuals with Serious Mental Illness

**DOI:** 10.3934/publichealth.2015.2.163

**Published:** 2015-04-22

**Authors:** Andrea M. Auxier, Bonni D. Hopkins, Anne E. Reins

**Affiliations:** Beacon Health Options, 200 State Street, Suite 302, Boston, MA 02109, USA

**Keywords:** Medicaid health homes, behavioral health homes, integrated care

## Abstract

Changes to the health care market associated with the Patient Protection and Affordable Care Act (ACA) are creating both need and opportunity for states, health plans, and providers to improve quality, outcomes, and satisfaction through better integration of traditionally separate health care delivery systems. Applications of the term “integrated care” vary widely and include, but are not limited to, the integration of care for Medicare-Medicaid dually enrolled beneficiaries, the integration of mental health and substance abuse (also known as behavioral health), and the integration of mental health and substance abuse with medical care, most commonly primary care. In this article, integrated care refers to well-coordinated physical health and behavioral health care. Medicaid Health Homes are emerging as a promising practice, with sixteen states having adopted the Health Home model through approved State Plan Amendments. This article describes one state's journey towards establishing Health Homes with a behavioral health focus. We discuss a partnership model between the relevant state organizations, the contracted providers, and the behavioral health managed care organization responsible for many of the supportive administrative functions. We highlight successes and operational challenges and offer recommendations for future Health Home development efforts.

## 1. Introduction: the clinical and economic impact of chronic illness among mentally ill Medicaid enrollees

Although exact statistics vary according to methodology, the clinical and economic burden of mental illness is well established in the literature. The largest surveys conducted to date estimate that mental illness affects between 18.6 and 22.5 percent of all U.S. civilian, non-institutionalized adults [1],[2]. Of these, 4.1 percent are classified as having a Severe Mental Illness (SMI), defined as a mental, behavioral, or emotional disorder (excluding developmental and substance use disorders) that is diagnosable currently or within the past year, of sufficient duration to meet diagnostic criteria specified within the 4th edition of the Diagnostic and Statistical Manual of Mental Disorders (DSM-IV), and that results in serious functional impairment that substantially interferes with or limits one or more major life activities [1]. Illicit drug dependence or abuse co-occurs in 11.6 percent of adults with SMI, 6.9 percent of adults with moderate mental illness, and 5.4 percent of adults with low (mild) mental illness [1].

Individuals with SMI have a higher average overall mortality rate and a lower life expectancy than those without SMI, dying 25 years earlier on average [3],[4]. The majority of excess deaths are due to medical illnesses, in particular cardiovascular disease, respiratory illness, and cancer [5]–[7]. Contributing factors to inadequate health care among the mentally ill are well documented and include health risk and lifestyle factors, medication side effects, and the direct cognitive, sensory, and social side effects of mental illness [8]–[11]. Although these factors create barriers to effective treatment, evidence suggests that there are also significant disparities in the provision of health care services among the mentally ill. For example, a review of 22 U.S. based studies revealed an 11 percent higher mortality rate in the year after acute heart disease for those with psychiatric diagnoses [12]. Although the highest number of excess deaths in schizophrenia is associated with cardiovascular disease, surgical interventions occur less frequently than in the general population [13],[14]. Additionally, people with psychotic disorders are less likely to receive routine cancer screening, standard levels of diabetes care, treatment for arthritis, and post-stroke treatment [15]–[18].

Medicaid expansion is resulting in millions of newly insured patients with a disproportionate share of comorbid medical and behavioral health conditions. Among nonelderly adult Medicaid enrollees in 2009, over a third (35%) had a diagnosed mental illness. Moreover, between 38% and 52% of those with diabetes, cardiovascular disease or respiratory disease also had a comorbid mental illness [19]. Behavioral health conditions are twice as prevalent as in the general population, health care costs are three and a half times as high, and hospitalization rates are four times as high due to co-occurring mental illness or substance use disorders [20],[21]. In 2002, more than half of disabled Medicaid enrollees with psychiatric conditions also had claims for diabetes and cardiovascular disease [20]. The top five percent (5%) of enrollees with the highest cost of care account for 50% of the total spend. Three of the five most prevalent disease pairs in this group include psychiatric illness, with the most common disease pair being cardiovascular and behavioral health illness. Forty percent of Medicaid enrollees in this top five percent (5%) have this illness combination.

From 2009 to 2011, average annual direct spending to treat mental health disorders in adults ages 18 to 64 totaled $48.2 billion, of which 24.2% was covered by Medicaid [22]. However, the additional medical costs incurred by this population total over $82 billion and are 2–3 times as high as those enrollees without comorbid mental health or substance use disorders [23]. Table 1 summarizes the breakdown of Medicaid health care spending for individuals with and without mental health or substance use disorders. Of note, the table illustrates that individuals with SMI have a much higher proportion of their total cost of care attributable to behavioral health needs than individuals without a mental health or substance use disorder. Nevertheless, medical costs for this population remain over twice as high as behavioral health costs, emphasizing the need for better integration of behavioral and medical care.

To achieve optimal cost savings for individuals with SMI, both behavioral and medical issues must be addressed through clinical approaches that recognize not only comorbidity, but also the mutually reinforcing role that behavioral health conditions have on medical conditions, and vice-versa. Based on their analysis, the authors of this report estimate that effective integration of behavioral health care with medical services can result in potential annual Medicaid cost savings of $7 to $10 billion. Savings potential ranges from $336 to $1,584 per member per month (PMPM), depending on condition.

**Table 1.** Total Medicaid health care spending by population and presence of behavioral conditions – 2012 costs (millions) [23]

Behavioral Health Diagnosis	Medical	Behavioral	Medical Rx	Behavioral Rx	Total
No MH/SUD	$134,920	$1,963	$27,710	$2,176	$166,769
MH/SUD	$82,655	$31,264	$18,759	$9,389	$142,067
Total	$217,575	$33,227	$46,468	$11,566	$308,836

## 2. Barriers to effective integrated care delivery for individuals with severe and persistent mental illness

Many people with mental health concerns, including but not limited to SMI, prefer to be seen in a primary care setting. Reasons include being more comfortable in a medical setting that does not specialize in mental illness, the convenience of receiving health care in a single treatment setting, and a preference for knowing that their behavioral health and primary care providers are working together. However, access to primary care continues to be an issue and most people with SMI are seen by specialty care, not primary care providers. For example, individuals with psychotic disorders and bipolar disorder are 45 percent and 26 percent less likely, respectively, to have a primary care doctor than those without mental disorders [24].

Numerous studies have demonstrated that primary care-based integrated services can enhance quality of care, decrease health care costs, improve overall health, decrease the burden on primary care providers (PCPs), improve PCPs' ability to address patients' behavioral health needs, and result in higher treatment initiation rates for behavioral health concerns [25]–[29]. While significant, most rigorous studies have limited the inclusion criteria to depression and anxiety, two of the most common behavioral disorders encountered in primary care settings. The seminal publication on the state of the integration of mental health and substance abuse and primary care was published in 2008 by the Agency for Healthcare Research and Quality (AHRQ). The document, a meta-analysis of randomized clinical trials and quasi-experimental design studies, concluded that while most behavioral health interventions in primary care settings were effective, particularly for depression, there was “no discernible effect of integration level, processes of care, or combination, on patient outcomes” [30]. A more recent review of the literature called for continued research with a shift from protocol-driven randomized trials focusing on depression to rigorous evaluation of non-disease-specific models [31].

Although over half of all mental health treatment in the U.S. is now delivered in primary care settings, the intensity and quality of treatment varies. Many cases go unrecognized and untreated, with only one-third of cases seen in the primary care sector receiving minimally adequate care [32]. Of people with behavioral health concerns, those with SMI are the most likely to receive suboptimal care in primary care settings. Reasons include PCPs not recognizing early signs of illness and/or being uncomfortable with mental illness, lack of availability or access to specialty mental health clinics, and poor treatment initiation rates among those referred to specialty mental health care clinics. Treatment initiation rates to specialty mental health are estimated to be less than 50% [33],[34]. The fact that most primary care practices have not yet adopted highly integrated models of care is also a significant barrier.

Given the challenges associated with delivering high-quality care to individuals with SMI, several initiatives have focused on integrating primary care services into behavioral health settings. Unlike the well-established literature detailing the benefits of integrating behavioral health into the primary care setting for depression and anxiety, the literature describing the integration of primary care into behavioral health settings is relatively new. Recently, the Milbank Memorial Fund conducted an extensive literature search and reported on findings derived from twelve randomized controlled trials. In general, integrated care, as well as care enhanced by trained nurse care managers, improved mental health-related outcomes and use of preventive and medical services as well as reduced cardiovascular risk factors in individuals with diabetes [35].

Other efforts have had mixed results. For example, an evaluation of the Substance Abuse and Mental Health Services Administration's (SAMHSA's) Primary and Behavioral Health Care Integration (PBCHI) program demonstrated improvements in indicators for diabetes, hypertension, and dyslipidemia, but no improvements in smoking or obesity [36]. There was also no clear connection between integrated care and behavioral health outcomes, though it is important to note that PBCHI did not target behavioral health outcomes. Similarly, results from Pennsylvania's Behavioral Health Home Plus program described early implementation challenges and emphasized the need for adequate investment in staff training and resources as well as enough time to allow for trial and error learning [37].

## 3. Defining Medicaid Health Homes

In contrast to concepts such as medical homes, health homes, and the Patient-Centered Medical Home™ (PCMH) [38], Medicaid Health Homes are specifically defined within Section 2703 of the Patient Protection and Affordable Care Act [39]. Medical homes and health homes are generic terms often used interchangeably to describe an integrated treatment philosophy, while PCMHs and Medicaid Health Homes are structured approaches that follow specified service-delivery guidelines. Although both place a strong emphasis on care coordination, continuous quality improvement, and population health management supported by information technology, critical differences exist. Table 2 summarizes distinguishing features of PCMHs and Medicaid Health Homes (hereafter referred to as Health Homes).

Health Homes offer states the option to implement certain programs for individuals with chronic conditions using Medicaid funding, with a 90 percent enhanced Federal Medical Assistance Percentage rate for the first eight fiscal quarters after approval of a Health Home State Plan Amendment (SPA). After receiving Centers for Medicare and Medicaid Services (CMS) approval for an SPA, a state Health Home may serve people with 1) two chronic conditions, or 2) one chronic condition and at risk for an additional chronic condition, or 3) a serious and persistent mental illness. The law defines a chronic condition as a mental health condition, substance use disorder, asthma, diabetes, heart disease, or being overweight (as evidenced by having a Body Mass Index over 25).

**Table 2.** Patient-centered medical homes and Medicaid Health Homes key dimensions.

Dimension	PCMH	Medicaid Health Home
Population Served	All	Specific chronic conditions
Designation	NCQA	State
Guidelines	Accrediting agency	ACA Section 2703
Payor Source	Multiple	Medicaid
Location	Actual clinic location	Actual clinic location or group of providers practicing across multiple settings
Scope	Physician-led primary care team coordinates overall health care needs	Can include primary care, community mental health, and ancillary support agencies

States have flexibility in determining what organizations can be a Health Home provider, but all Health Home providers must coordinate and provide access to preventive services, mental health and substance abuse services, comprehensive care management and care coordination, disease management, and long-term supports. Of note, states are required to consult with SAMHSA about how they plan to provide mental health and substance use disorder treatment, regardless of the focus conditions chosen [40]. Health Homes do not need to provide all required services directly; however, they must ensure that the following services are available and coordinated:

1. Comprehensive care management
2. Care coordination and health promotion
3. Comprehensive transitional care, including appropriate follow-up, from inpatient to other settings
4. Enrollee and family support (including authorized representatives)
5. Referral to community and social support services, if relevant
6. Use of health information technology to link services, as feasible and appropriate

## 4. Status of state Medicaid Health Homes

According to the CMS Health Home Information Center, as of December 2014, 16 states have received approval from CMS to embark upon the Health Home program through 21 State Plan Amendments with a current enrollment of 1,046,508 [41],[42]. Early programs included those in Rhode Island, North Carolina, and Oregon and later programs have included those in Alabama, Idaho, Iowa, Kansas, Maine, Maryland, Missouri, New York, Ohio, South Dakota, Vermont, Washington, and Wisconsin. While most states have elected broad focus areas consisting of chronic conditions and SMI and/or substance use disorders (SUD), six states (Iowa, Kansas, Maryland, Missouri, Ohio, and Rhode Island) have approved SPAs specifically for Health Homes with a mental health orientation, with a current enrollment of 56,056. Maine has a two-staged approach that includes a focus on management of individuals with SMI in stage B, Illinois and Connecticut have submitted SPAs pending approval, and Virginia has announced plans to submit a SPA for behavioral Health Homes for 13,000 adults and children with SMI. Two states (Rhode Island and Vermont) have approved SPAs focused on individuals with SUD.

## 5. Medicaid Health Home challenges

Although CMS has issued guidance for Health Homes in the form of two State Medicaid Director Letters [43], states have identified many areas of interpretation, such as data collection, reporting, and quality control as they have implemented their programs. As states are in varying stages of seeking approval for SPAs and are only now in the process of implementing their Health Homes programs, data and published studies are sparse. The most definitive information available to date is the Interim Report to Congress on the Medicaid Health Home State Plan Option issued by the Department of Health and Human Services, Office of the Secretary [44]. To provide a context for the discussion of a specific state's program, we have identified from the report several challenges that many states are confronting, including:

- Serving both children and adults in a Health Home program—the program requires coverage for both adults and children; states have responded by using providers that serve a specific population
- Defining targeted case management—states have found it challenging to integrate existing state requirements for targeted case management within the Health Home program, without duplicating care management services
- Enrolling individuals—identifying and enrolling high-need individuals is a pervasive challenge for the Health Homes programs; states have responded by using technology to assign a risk rating to identify and prioritize individuals for enrollment
- Coordinating with Managed Care Organizations (MCOs)—coordination challenges between Health Homes and MCOs have necessitated that states define and clarify roles and responsibilities for each participating organization
- Integrating Health Information Technology (HIT)—states are in various stages of implementation of their HIT systems, which has presented challenges for Health Home care coordination as well as data reporting requirements
- Care transitions—among other examples of coordination, states are relying upon alerts and notifications to ensure that individuals that present to the emergency room are receiving follow up services from Health Home providers
- Provider Administrative Burden—Health Home providers have voiced concern over the attestation and billing requirements for the program; in response, certain states have implemented alternate payment methods

## 6. Connecticut's Behavioral Health Home initiative

Several states are conceptualizing and developing Health Home programs focused on supporting the needs of individuals experiencing a Serious Mental Illness. The State of Connecticut is profiled to demonstrate the challenges inherent in this type of system change.

The Connecticut Medicaid behavioral health system is supported by an Administrative Services Organization (ASO) known as the Connecticut Behavioral Health Partnership (CTBHP). The Partnership comprises the Department of Mental Health and Addiction Services (DMHAS), the Department of Children and Families (DCF), the Department of Social Services (DSS), and ValueOptions, a national managed behavioral health organization^1^. The Partnership was designed to create an integrated behavioral health service system for Connecticut's Medicaid populations and is overseen by a legislatively mandated Behavioral Health Partnership Oversight Council.

The Partnership's goal is to provide access to a more complete, coordinated, and effective system of community-based behavioral health services and support. In August 2012, the Adult Quality, Access and Policy sub-committee of the Behavioral Health Partnership Oversight Council formed a Behavioral Health Home (BHH) workgroup to outline the contractual and operational specifications for the BHH Program [45]. This workgroup was tasked with:

1. Establishing parameters for defining eligibility for the BHH
2. Establishing service definitions
3. Identifying provider standards
4. Identifying outcome measures
5. Reviewing Medicaid and DMHAS enrollment data

The result of this work was the development of the BHH model to bridge the gap between behavioral health and primary care for individuals with SMI. The goals of the model are:

1. Achieve the Triple Aim of improving individual experience of care, improve population health, and reduce per capita health care costs
2. Implement and evaluate the BHH as a way to achieve accessible, high quality integrated health care
3. Demonstrate cost-effectiveness to justify and support the sustainability and spread of the model
4. Support behavioral health practice sites by increasing available primary care resources and enhancing care coordination to result in improved quality of care and patient outcomes
5. Decrease unnecessary inpatient hospitalization and emergency room visits

DMHAS determined that the most expedient way to implement statewide BHH services for the targeted population was to transform and expand the current behavioral health system to include an array of inter-disciplinary behavioral health services, medical care, and community-based social services and supports [46]. Thirteen existing Local Mental Health Authorities (LMHAs) and their affiliated provider networks created a natural framework and infrastructure on which to integrate primary care and associated BHH services. The LMHAs are designated by the DMHAS and each has responsibility for providing services within a specified catchment area, assuring statewide coverage.

To become a BHH, each LMHA was required to:

- Meet state credentialing requirements/BHH eligibility standards
- Enhance their existing staffing to ensure capacity to provide Health Home services (Care Managers, Primary Care Consultants, Transition Coordinators, Peer Recovery Specialists, and others)
- Coordinate all behavioral health, physical health, and rehabilitation services in accordance with a comprehensive integrated care plan
- Commit to providing all six core services to enrollees
- Meet all State and Federal contracting and reporting requirements

In addition to the initial contracting of the BHHs directly by DMHAS, in January 2014, DMHAS released a Request for Proposal (RFP) for an ASO to supplement and enhance the State's administrative infrastructure to implement this program successfully, with an emphasis on process and outcomes data evaluations. The ASO is specifically contracted to perform a variety of critical functions in support of DMHAS's recovery-oriented BHH services, including communication with providers regarding data to support and drive positive outcomes. These functions are described in Table 3.

**Table 3.** Behavioral health home administrative service organization critical functions [47].

Function	Tasks
**Provider Credentialing**	Develop credentialing application based on provider requirements
	Manage the credentialing process
	Assistance with contracting
**Provider Training and Member/Provider Relations**	Develop learning community for providers
	Provider direct customer assistance through a call center
	Manage complaints and grievances
	On and off-site training of providers
	Conduct site visits and audits
**Data Analytics and Enrollment**	Conduct data analysis to target enrollment based on eligibility information
	Attribute eligible individuals to Health Homes
	Support notification of attribution and opportunity to opt-out
	Track eligible individuals through their BHH enrollment
**Health Information Technology Development**	Develop a Health Insurance Portability and Accountability Act (HIPAA) compliant web-based electronic health record (EHR) for providers to input and retrieve data
	Data to include BHH services, medical and pharmacy services, authorization and claims, assessment and recovery planning, quality/outcome measures
	Member, provider, and client portals with rules-based secure access to data
**Data Management and Reporting**	Collection and synthesis of data to include outcomes/quality measures, productivity, individuals served, inpatient and emergency department (ED) utilization, Medicaid claims analysis, and other information
	Development of reports based on data collection for providers, DMHAS, and CMS

## 7. Connecticut Partners for Integrated Care

Building on the successful administration of mental health and addiction services for the CTBHP, DMHAS awarded the BHH ASO to ValueOptions in April 2014, although the final contract is still being approved by the state and the State Plan Amendment has not yet been approved by CMS. ValueOptions will be operating the Behavioral Health Home program through a contractual relationship with Advanced Behavioral Health (ABH) of Connecticut as the Connecticut Partners for Integrated Care (the Partners). ABH was founded in Connecticut in 1995 as a non-profit organization to manage Connecticut public sector behavioral health care programs. Due to their current behavioral health and social services program management and longstanding professional relationships with providers and stakeholders, including DMHAS, the Partners leverage the complementary experience and expertise of both organizations to be a resource to DMHAS, providers, enrollees, and other constituents in managing the BHH system.

Across the contracting phase, it has been helpful to have multiple entities at the table to draw upon prior successful joint implementations. For example, all entities are involved in designing performance target metrics and in establishing work flows for new information technology processes. Mutual trust and respect have been critical during the contracting phase, as flexibility is needed to allow the state to hone its needs assessment, procedures, and expectations.

## 8. Connecticut's Behavioral Health Home population

Connecticut defined its population for the Health Home program as “a Medicaid recipient who has been diagnosed with a Serious and Persistent Mental Illness (SPMI), defined as Schizophrenia and Psychotic Disorders (295.1–295.35, 295.60–295.75, 295.9x, or 297.1), Mood Disorders (296.0x, 296.3–296.6, or 296.89), Anxiety (300.21–300.23), Obsessive-compulsive Disorder (300.3), Borderline Personality Disorder (301.83), and Post Traumatic Stress Disorder (309.81), and has combined Medicaid claims (for medical and behavioral health services), which exceed $10,000 in a calendar year” [Draft Personal Service Agreement between Connecticut and ValueOptions, Inc.].

At this point in the implementation, the State has shared the list of identified members already receiving services at each BHH, while other members are not yet affiliated. There have been challenges in determining the most efficient method of enrolling affiliated members in the BHH, as well as the process for outreach and enrollment for members not yet connected to the LMHA. In addition, while the SPMI diagnosis is quite familiar to the BHHs, the medical costs and related services that resulted in inclusion are not yet fully understood by providers.

As the implementation process continues to unfold, it has become clear that the identified members may be difficult to enroll and that additional time and support will be needed for this process. As the ASO becomes more involved after the contract execution, we will be tracking enrollment and monitoring for potential barriers such as capacity to engage members who were not previously affiliated. Prior to establishing the contract, the volume of members not yet connected to the LMHAs had not been anticipated by the state and processes to handle all aspects of the program implementation for these members continues to develop. Currently the estimates are 6,500 affiliated and 20,000 unaffiliated members.

## 9. Performance outcome measures

In addition to the CMS Technical Specifications for Health Homes [Centers for Medicare & Medicaid Services (CMS), Core Set of Health Care Quality Measures for Medicaid Health Home Programs, Technical Specifications and Resource Manual for Federal Fiscal Year 2013 Reporting, dated March 2014. Available from: http://www.medicaid.gov/State-Resource-Center/Medicaid-State-Technical-Assistance/Health-Homes-Technical-Assistance/Downloads/Health-home-core-set-manual.pdf] program performance will be assessed through additional Connecticut-specific measures upon CMS approval of the SPA and a signed ASO contract.

## 10. Medicaid behavioral Health Home development challenges

A significant hurdle in Health Home implementation is the innovative nature of the programs themselves. Currently there is large-scale adoption of integrated health home models across the country with limited state experience or lessons learned to build upon at this scale. Despite continued academic research and best-practice pilot programs, each state is essentially adopting an individual approach by learning what works best at the systems level and adapting their models for their specific circumstances. Although CMS has issued guidance for Health Homes in the form of two State Medicaid Director Letters [43], states have identified many areas of interpretation, such as data collection, reporting, and quality control as they have implemented their programs. Based upon review of the literature and the experience of the current vendors in Connecticut, the following are challenges and lessons learned while developing a system of care with a Health Home foundation as the principal delivery mechanism for Medicaid-funded medical and behavioral health services.

### 10.1. Serving both children and adults

Although complex care conditions are defined in the legislation, the Health Home statute does not allow states to limit program eligibility to a specific age range or population category (e.g., children, adolescents, and dual eligibles must be included). This lack of ability to tailor the program to a particular population presents several design challenges, including how to find and enroll members, which providers and social supports will serve them, how best to define outcome measures useful for both child-serving and adult-serving systems. Instead of delimiting their programs through age exclusions, CMS encourages state Medicaid agencies to focus their Health Home programs to target certain conditions that are prevalent in a particular age group or to contract with providers that have historically served members of certain age categories [48].

Various states have responded by using specific providers that focus on serving a specific population. Other states have required safety net providers to expand their service offerings while continuing to treat both adults and children. For behavioral Health Homes specifically, the definition of a serious mental illness is often restricted to an adult population. In Connecticut, the State is expanding the behavioral Health Home contractual requirements to include children and families experiencing a Serious Emotional Disturbance (SED) in addition to SMI adults, particularly when those children are already served by the LMHAs that are becoming BHHs.

### 10.2. Achieving comprehensive care management

Integrating existing state requirements for certain services into Health Home programs is not straightforward. Providers have voiced concern over the attestation and billing requirements possibly resulting in additional administrative burden and cost shifting as opposed to true practice transformation; for example, targeted case management is a significant source of ongoing revenue for existing specialty safety net providers. Eligibility for targeted case management is determined by functional impairment and intervention focuses exclusively on needs that relate to that functional impairment. While this level of support is necessary, it can fall short of Health Home expectations regarding care coordination and care management. States are therefore faced with having to develop solutions that ensure that care coordination and care management provided by the Health Home is comparable to the targeted case management they replace.

States have sought to expand care management activities to encompass a broader examination of the social determinants of health, including the entire array of individual biopsychosocial needs. States have accomplished this expansion through a variety of means, including initially requiring Health Homes to contract with the existing care management entities or safety net providers for targeted case management, then gradually scaling up care management activities by requiring the Health Home to provide care management that addresses not only standard areas typically addressed by targeted case management but additional measures, and by contracting with the existing specialty safety net providers to become Health Homes. With all of these solutions, it is incumbent upon the State (or contracted vendor) to ensure that contractual expectations for comprehensive care management are being met.

### 10.3. Streamlining processes and recognizing administrative complexity

As States adopt Health Home models as the primary delivery mechanism for Medicaid-funded integrated care, the resources and administrative infrastructure available to support this system transformation are as variable as each state program. Several states, recognizing these challenges, are contracting with an Administrative Services Organization (e.g., Connecticut) or Managed Care Organizations (e.g., Kansas, New York) to support providers as they make this significant transition. In most cases, the desire to move to a Health Home model, as well as legislative approval and funding of these models, has preceded an honest assessment of the administrative requirements and resources necessary for deployment of a successful program. Underestimating administrative complexity can result in “scope creep,” including expansion of roles, responsibilities, and allocation of resources to meet the needs of the BHH. The associated delays create issues between state contract expectations for program development, particularly around information technology and reporting systems, and the vendor's time and financial constraints. Particularly during the implementation phase, it is difficult to align the execution of multiple contracts and the SPA, creating additional pressure on the system to compensate. Moreover, as the BHH is operationalized, the magnitude of system needs and advancements in the field often evolve beyond initial contracts.

### 10.4. Using Health Information Technology (HIT) to coordinate with existing health delivery systems

Health Information Technology across health care delivery systems (e.g., primary care, behavioral health, inpatient, and long term care) is essential for achieving a truly coordinated system. For example, HIT can be used to ensure that individuals who present to the emergency room are receiving follow up services from Health Home providers, to facilitate scheduling referral appointments in real time, to send appointments reminders, to alert the care team if high-risk clients miss appointments or do not refill critical prescriptions, and to automate information sharing between providers.

Despite the promise of HIT, barriers to achieving full functionality remain. First, states are in various stages of implementation of their HIT and Health Information Exchange (HIE) systems, making it difficult to meet Health Home reporting requirements. Second, new technology is not always adopted even when it is available, due to financial and operational considerations. Practices that can finance information technology enhancements are not always convinced that the return on investment will justify the expense. There are additional financial and other expenses that are incurred. Specifically, employees need to be trained on how to use the technology, and workflows and associated protocols need to be established. Additionally, behavioral health providers are not eligible for Meaningful Use incentives to offset the cost of EHRs or the provider licensing fees that some EHRs require. Any information gleaned from new technology must also be incorporated into existing quality improvement programs so that it can yield practice change. Although often desired, practice change can disrupt a practice's routine operations. There is also concern among providers about the current capacity of EHRs to manage consents and re-disclosure of substance use data.

### 10.5. Including all relevant parties

By definition, Health Homes are intended to promote better access and improved care for individuals with chronic conditions, with attention to medical, behavioral, and social support needs. However, provider entities that are designated as Health Homes must work with other providers and agencies that are typically neither specified in the contract nor incentivized to collaborate with the Health Home. For example, Behavioral Health Homes may be expected to secure medical care for enrollees, to provide outreach and education regarding mental illness to primary care providers, and to establish protocols and mechanisms to ensure information exchange with providers who are not included in the contract.

In Connecticut, the Connecticut Partners for Integrated Care (the Partners), which is headed by the current behavioral health Administrative Services Organization, ValueOptions, is only directly contracted with the state to support the Health Home initiative, including oversight of the BHHs. Consequently, there will be a need for considerable collaboration with the BHHs themselves, the Medical ASO, and the medical providers. However, the state must still work with all parties to specify which entity is ultimately responsible for meeting members' care coordination needs, including medical care coordination. Moreover, CMS Health Home Outcome specifications sometimes include information not readily available through claims or BHH records. To support engaging PCPs in BHHs, the Partners will be reaching out to all Medicaid medical and dental providers to provide education regarding behavioral health issues, and to promote inclusion of members with behavioral health concerns in their practices. The state and the Partners will also support the BHHs in securing memoranda of understanding with medical providers to share information, coordinate care, and seek necessary system changes to support this collaboration. Beyond offering financial incentives to PCPs, payers have incorporated and supported PCPs in Health Homes through a variety of means, including offering toll-free access lines to psychiatrists and other specialists for pediatric consultations, training on behavioral health topics, setting up information technology systems to offer PCPs triggers and reminders for member medication management, promoting telehealth strategies, and building information exchanges between specialists and PCPs through secure information technology platforms, as well as other means [49].

### 10.6. Enrolling individuals

Identifying and enrolling high-need individuals can pose significant challenges for Health Home programs. Heath Homes must have the capacity and willingness to serve everyone who presents, including those Medicaid members that have traditionally had less access to primary care (e.g., individuals with significant mental illness, substance use disorders, developmental disabilities, and other conditions). Additionally, the members must be allowed a choice of providers. While desirable, this can compromise continuity of care, particularly with those primary care and specialty safety net providers who are not going to become a Health Home and who have longstanding relationships with the member. Moreover, additional research is needed to determine the optimal thresholds for identification of appropriate individuals based on prior claims cost to ensure impactible chronic illnesses benefit from the focus of care coordination.

## 11. Discussion

The road towards establishing a statewide Health Home-supported system of care can be both exciting and daunting. While the benefits of successful implementation are increasingly clear, operational hurdles remain. The Health Home concept is not based on new ideas, the structure of the Health Home as defined in the ACA is a recent development. Because large-scale transformation initiatives are complex and iterative, it will take time to determine best-practice solutions to implementation. Health Homes provide an opportunity for payers, providers, and managed care organizations to address care delivery challenges by building upon the lessons learned from prior programs. We offer the following recommendations with the aim that they will be useful to readers involved in future Health Home efforts.

### 11.1. Making room for children in the Health Home

### 11.2. Assessing parallel efforts

As noted earlier, Health Homes rely on collaboration between multiple systems with clear roles and responsibilities, some of which may not be specified in associated contracts. For this reason, it is important to determine any concurrent efforts that may share the same the goals as the Health Homes and/or where collaboration may be of mutual benefit. In the case of a Behavioral Health Home, it might be beneficial to explore the possibility of including primary care providers in the Health Home contract. As a corollary to this recommendation, we emphasize that much of the work involved in constructing a Health Home program will fall outside of the scope of the contract and will be relational in nature. Leveraging existing community relationships and fostering new ones that align with the Health Home mission will be critical to successful outcomes.

### 11.3. Determining requirements and specifications

While vendors can bring significant resources and expertise in systems development, business requirements and specifications must be fully developed and defined prior to engagement. Additionally, with the limited budgets available to provide administrative supports, states will be required to temper their expectations regarding the level of sophistication that can be achieved within the desired time frames.

### 11.4. Maximizing meaningful use of HIT

To be effective, HIT must allow the entire treatment team to enter and retrieve member information for treatment planning, initial encounter tracking, follow-up care encounter tracking, and care transitions. The data must also be reportable so that it can be analyzed at both the individual and aggregate levels. Even when technology is available, practices do not always have workflows and systems in place to ensure that data is captured consistently. Additional releases of information may need to be developed and integrated into existing workflows, particularly to satisfy 42 CFR requirements for disclosing substance use.

Unclear expectations about who is responsible for delivering the services and how they are to be documented in the clinical record can lead to reporting errors that compromise data validity. A common example is smoking cessation and counseling. Because physicians, behavioral health providers, and medical support staff all may deliver the service, it is often duplicated and/or captured in different parts of the medical record. Additionally, smoking is not routinely coded as a primary diagnosis or billed through the proper CPT code. In many instances, tobacco screening and cessation are recorded as narrative text in lieu of structured fields, making reporting difficult and often inaccurate. Troubleshooting these issues as they arise will help Health Home staff function more effectively as a team and will help prevent the use of inaccurate data in clinical decision-making.

### 11.5. Considering the integration continuum

Given the clinical and economic burden associated with fragmented care and an increasing number of reform initiatives, a growing number of practices are turning towards integrated models to address the complexities of co-occurring disorders. However, inconsistent definitions about what constitutes “integration” can contribute to confusion at the practice level. While Health Home legislation requires coordination between providers working in different settings, it does not require providers to be co-located in the same setting.

Where possible, we recommend that Health Homes consider co-located arrangements. Co-location allows for a single repository for a broad range of mental health, substance abuse, and medical problems, additionally allowing for relationships conducive to the sharing of medical and behavioral issues between primary care providers and behavioral health providers. Moreover, this decreases the burden for individuals in scheduling appointments with multiple providers, who often have transportation and other economic barriers to seeking care. Co-location can improve behavioral health treatment initiation rates in primary care settings and increase the likelihood that individuals with SMI will seek integrated services [28],[35]. Service-delivery models that include co-location can range from a single provider operating independently from the treatment team to highly integrated systems where both kinds of providers work collaboratively, with open communication regarding patients supported by robust operational and administrative infrastructure. Integration frameworks such as the Standard Framework for Integrated Health Care and the Lexicon for Behavioral Health and Primary Care Integration aim to define the characteristics of what it means to be “integrated” [48],[49]. In 2014, two public domain measurement instruments corresponding to each of these frameworks were developed, the Integrated Practice Assessment Tool (IPAT) and the Vermont Integration Profile (VIP [52],[53]. States can use these instruments to determine how integrated a practice is, to monitor progress along the integration continuum, for comparative analysis, to examine network readiness for integration, to establish thresholds for differential reimbursement, or to tailor technical assistance programs to a practice's needs. In addition, tools such as the IPAT help normalize the process of moving along a continuum of integrated care and inspire the undertaking of system transformation.

Despite the potential benefits of co-location, it is important to note that co-location does not ensure practice transformation. One of the main lessons learned from integration efforts is that simply co-locating staff does not necessarily lead to improved outcomes. Providers and care managers require training and ongoing support to be able to function as a collaborative care team, defined as “an approach to integration in which primary care providers, care managers, and psychiatric consultants work together to provide care and monitor patients' progress” [54]. The key collaborative care components of integrated care that have been associated with reduced costs, improved outcomes, and greater satisfaction among providers and patients with depression and anxiety in primary care are [55]:

1. Self-care support
2. Care management and care team responsible for care
3. Treatment to target (systematic tracking of disease severity) and outcomes measurement
4. Stepped care: Care provided is least extensive, intensive, and expensive needed for positive outcomes; intensity stepped up if no improvements
5. Systematic caseload review, consultation and referral
6. Patient tracking and registry functions
7. Adoption of evidence-based interventions/guidelines
8. Engagement of social service agencies

Behavioral Health Homes provide an important testing ground for the effectiveness of integrated collaborative care specifically for individuals with SMI. Rather than adopting any one integrated care model, the Institute for Healthcare Improvement suggests selecting collaborative care components that fit with patient needs and organizational characteristics, and then investing in continuous quality improvement efforts to operationalize these components [56].

## Footnotes

1 On December 23, 2014, ValueOptions' parent company, FHC Health Systems, Inc. (FHC) merged with Beacon Health Vista Merger Sub, Inc. and became a wholly owned subsidiary of Beacon Health Vista Parent, Inc.
